# Association Between the Levothyroxine Therapy and the Levels of Anti–Thyroid Antibodies in Women with Hypothyroidism Due to Hashimoto’s Thyroiditis—Narrative Review

**DOI:** 10.3390/ijms27114864

**Published:** 2026-05-28

**Authors:** Karolina Wrońska, Urszula Szczuko, Iwona Szydłowska, Jolanta Nawrocka-Rutkowska, Leon Rudak, Lidia Kwiatkowska, Iga Szukalska, Małgorzata Szczuko

**Affiliations:** 1 Department of Bromatology and Nutritional Diagnostics, Pomeranian Medical University in Szczecin, 70-111 Szczecin, Poland; urszula.szczuko@gmail.com (U.S.); leon.rudak88@gmail.com (L.R.); lidia.kwiatkowska98@gmail.com (L.K.); 73523@student.pum.edu.pl (I.S.); malgorzata.szczuko@pum.edu.pl (M.S.); 2 Department of Gynecology, Endocrinology and Gynecological Oncology, Pomeranian Medical University in Szczecin, 71-252 Szczecin, Poland; iwona.szydlowska@pum.edu.pl (I.S.); jolanta.nawrocka.rutkowska@pum.edu.pl (J.N.-R.)

**Keywords:** Hashimoto’s thyroiditis, antibodies, levothyroxine, anti–TPO, anti–TG, inflammation, treatment, pregnancy

## Abstract

Hashimoto’s thyroiditis (HT) is one of the most common autoimmune disorders with a complex etiology and pathogenesis, representing a significant challenge for medicine. Current pharmacological management of HT focuses on the treatment of hypothyroidism rather than addressing the underlying cause. Available scientific evidence provides limited and inconsistent data regarding the association between levothyroxine (L–T4), its dosage, and the levels of antibodies against thyroid peroxidase (anti–TPO) and thyroglobulin (anti–TG) in women with HT. Therefore, the aim of the study was to summarize current findings in this field. This narrative review was conducted using elements of the PRISMA 2020 guidelines. Literature analysis was performed using EMBASE, SCOPUS, and PUBMED databases. Articles published within the last 20 years were included. The search terms included “Hashimoto’s thyroiditis”, “Hashimoto’s disease”, “levothyroxine”, “L–thyroxine”, “anti–TPO”, “anti–TG”, “anti–thyroid antibodies”. Furthermore, to obtain a comprehensive overview of the available knowledge, the search terms were combined with “inflammation,” “treatment,” and “pregnancy”. Treatment with levothyroxine may potentially lead to a decrease in the levels of thyroid antibodies, primarily anti–TPO, in hypothyroid women with HT. Nevertheless, the direct effect of L–T4 on anti–thyroid antibody levels remains inconclusive and varies among individuals. Treatment with L–T4 in hypothyroid women with HT was also associated with improved fertility and pregnancy outcomes. The findings summarized in this review may contribute to systematizing current knowledge regarding L–T4 therapy in women with HT and its potential benefits related to fertility. Further clinical studies are required, particularly those addressing the immunological mechanisms of levothyroxine action, which may improve therapeutic precision in HT.

## 1. Introduction

Chronic lymphocytic thyroiditis (Hashimoto’s thyroiditis, HT) is one of the most common autoimmune thyroid diseases (AITD), affecting approximately 5–10% of the general population [1]. The etiology of the disease is multifactorial and not fully understood, involving genetic, environmental, and epigenetic factors [2]. Genetic susceptibility plays a vital role in the development of Hashimoto’s thyroiditis [3]. Scientific evidence indicates that HT may occur in individuals with polymorphisms in genes involved in immune regulation, including human leukocyte antigens (HLA), protein tyrosine phosphatase non–receptor type 22 (PTPN22), interleukin 1 receptor antagonist (IL1RN), interleukin 1β (IL1β), interleukin 17F (IL17F), glucocorticoid–induced TNFR–related gene (GITR), and signal transducer and activator of transcription 3 (STAT3) [3]. Polymorphisms in the cytotoxic T–lymphocyte–associated protein 4 (CTLA–4) gene, which encodes an immune regulatory protein involved in peripheral tolerance and T–cell function, have also been associated with HT [4]. The pathogenesis of HT is determined by immune system dysfunction, characterized by infiltration of autoreactive lymphocytes and loss of immune tolerance to self–antigens. Autoimmune activity in HT is associated with the production of antibodies against thyroid peroxidase (anti–TPO) and thyroglobulin (anti–TG) [5]. Anti–TPO antibodies are found in around 90–95% of people with Hashimoto’s thyroiditis and may cause oxidative stress. Anti–TG antibodies are less common in Hashimoto’s thyroiditis than anti–TPO antibodies, and their presence may be linked to the massive release of thyroglobulin following damage to thyroid tissue, as a result of thyroiditis, tissue injury, or surgery involving the gland, as well as due to iodine excess [6]. In addition to humoral immune responses, cellular immunity plays a crucial role, with infiltration of the thyroid gland by T lymphocytes. T helper cells (Th), including Th1 and Th17 subsets, as well as regulatory T cells (Treg), are particularly vital. An imbalance between Th17 and Treg cells contributes to disease progression [7]. Accumulation of pro–inflammatory cells results in thyroid damage and insufficient hormone production—thyroxine (T4) and triiodothyronine (T3) [8]. Consequently, patients require thyroid hormone replacement therapy, most commonly with levothyroxine (L–T4), and less frequently in combination with liothyronine (L–T3) [9]. Current diagnostic and therapeutic strategies focus primarily on early detection of hypothyroidism and initiation of hormone replacement therapy [10]. However, the immunological mechanisms underlying HT extend beyond the thyroid gland, affecting multiple organs and systems. Therefore, HT should be considered a systemic disease, and new therapeutic approaches targeting autoimmune processes are required [11]. Emerging evidence suggests that levothyroxine may also influence chronic inflammation in HT. Therefore, combining L–T4 with additional therapeutic strategies targeting autoimmune processes may be advantageous [12]. Moreover, it is essential to evaluate the impact of levothyroxine dosage not only on thyroid function but also on immunological and inflammatory parameters. The aim of this narrative review was to provide a synthesis of the extant literature on the association between L–T4 and the levels of anti–thyroid antibodies, as well as fertility in women with hypothyroidism and HT. The inconclusive findings of scientific studies on the effect of L–T4 on immunological parameters highlight a need to systematize existing knowledge and outline directions for future clinical research.

## 2. Materials and Methods

### 2.1. Search Strategy

This narrative review incorporated elements of the PRISMA (Preferred Reporting Items for Systematic Reviews and Meta-Analyses) guidelines to enhance the transparency, credibility, and scientific rigor of the review. The search strategy was aimed at gathering available and recent data on L-T4 therapy, its association with anti–thyroid antibodies, and fertility results in women with hypothyroidism and HT. The literature search strategy was developed in advance based on the PICO model, in order to clearly define the topic and identify the research area:

– Population (P): women with hypothyroidism and Hashimoto’s thyroiditis
– Intervention (I): levothyroxine therapy; additionally: combination therapy
– Comparator (C): lack of treatment for hypothyroidism in women with Hashimoto’s thyroiditis; various doses of levothyroxine
– Outcomes (O): anti–TPO and anti–TG antibody levels; additionally: inflammatory markers; fertility and pregnancy outcomes

The narrative review evaluates the above–mentioned topics, considering the literature published up to 26 March 2026. It examined the influence of L–T4 on anti–TPO and anti–TG titers, the association between antibody levels and L–T4 dose, and differences in treatment outcomes depending on disease severity and duration of treatment. The literature search was conducted in the PubMed, Embase, and Scopus databases. The search keywords were “Hashimoto’s thyroiditis”, “Hashimoto’s disease”, “levothyroxine”, “L–thyroxine”, “anti–TPO”, “anti–TG”, “anti–thyroid antibodies”.

The search was conducted using the following terms identified as medical subject headings (MeSH) and all field terms: 

PUBMED:

“Hashimoto’s thyroiditis” OR “Hashimoto’s disease” AND “levothyroxine” OR “L–thyroxine” AND “anti–TPO” AND “anti–TG” OR “anti–thyroid antibodies”

EMBASE: 

((‘hashimoto thyroiditis’/exp OR ‘hashimoto thyroiditis’ OR ‘hashimoto disease’/exp OR ‘hashimoto disease’) AND (‘levothyroxine’/exp OR ‘levothyroxine’) OR ‘l–thyroxine’/exp OR ‘l–thyroxine’) AND ‘anti–tpo’ AND ‘anti–tg’ OR ‘anti–thyroid antibodies’

SCOPUS: “Hashimoto’s thyroiditis” OR “Hashimoto’s disease” AND “levothyroxine” OR “L–thyroxine” AND “anti–TPO” AND “anti–TG” OR “anti–thyroid antibodies”

The terms were combined with “inflammation,” “treatment,” and “pregnancy” to provide an integrative approach to the subject under study. In order to avoid the possibility of overlooking vital sources and obtain a comprehensive view on the topic, a manual search of the reference lists of selected articles was also conducted. 

The analysis also included a study conducted by some of the authors of this review. The study met all inclusion criteria, and the article selection process was carried out by other independent authors of the review. A quantitative meta-analysis was considered during manuscript preparation; however, it was ultimately not performed due to substantial heterogeneity among studies regarding:

patient populations,

disease severity,

duration of levothyroxine therapy,

antibody measurement methods,

treatment protocols,

and reported outcomes.

Additionally, many studies lacked sufficient quantitative data necessary for reliable pooled statistical analysis.

### 2.2. Inclusion and Exclusion Criteria

**Figure 1:**
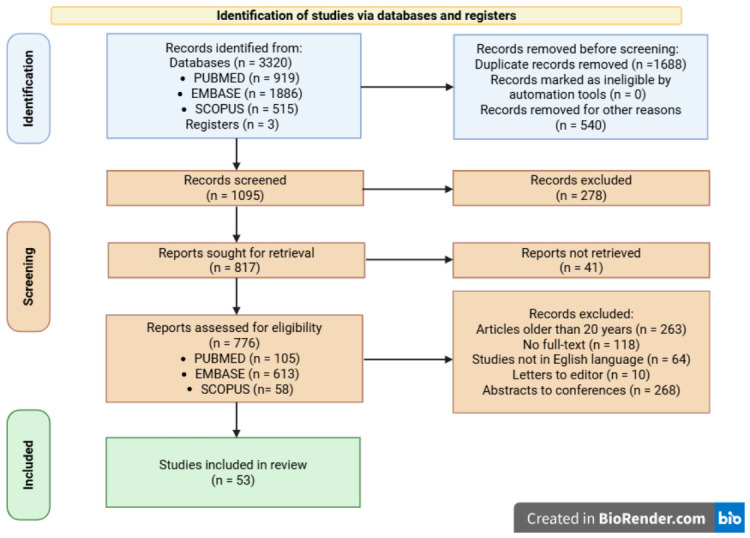
PRISMA Flow diagram. Created in BioRender. Wrońska K. (2026) https://app.biorender.com/illustrations/65f1ae58219dc6405d54874b.

This narrative review included 53 articles that met the inclusion criteria. The inclusion criteria comprised: original research articles, Clinical Trial, Clinical Study, Randomized Controlled Trial (RCT), Non–Randomized Studies (NRS)**,** Systematic Review, Review, full text, publication in English, and appearance in peer–reviewed scientific journals. Studies older than 20 years, non–English publications, letters to the editor, case studies, and conference abstracts were excluded, as shown in the flow chart in Figure 1. The exclusion criteria also included animal studies, retracted publications, articles lacking open access, and papers with incomplete or insufficient data. 

### 2.3. Data Extraction

The literature search was conducted independently by two authors, who also removed duplicate records identified across the three databases. In order to ensure methodological rigor and minimize bias, articles were selected according to a multi–stage procedure.

## 3. Results

### 3.1. Pharmacological Treatment of Hypothyroidism in Hashimoto’s Thyroiditis

Pharmacological treatment of women with hypothyroidism using levothyroxine alleviates the symptoms associated with hypothyroidism and helps to normalize thyroid–stimulating hormone (TSH) levels [9]. Hypothyroidism results in multiple clinical symptoms, including fatigue, weight gain, cold intolerance, and dry skin, necessitating thyroid hormone replacement therapy to restore metabolic function and improve quality of life [10]. HT also has a negative impact on female fertility and the course of pregnancy, contributing to difficulties in conceiving, an increased risk of recurrent miscarriages, fetal growth restriction, pre–eclampsia, and preterm birth; therefore, the introduction of L–T4 is of significant importance for women with HT who are planning a pregnancy [13]. Levothyroxine is typically administered orally in tablet form at doses of approximately 1.6–1.8 µg/kg/day, which are generally sufficient to normalize thyroid function. Nevertheless, in some patients with refractory hypothyroidism and persistently elevated TSH levels, higher doses exceeding 1.9 µg/kg/day may be required [14]. After administration in a fasting state, peak absorption of L–T4 occurs within approximately 90 minmin, with maximal serum concentrations reached after about 2 h. On average, 60–80% of the administered dose is absorbed and becomes bioavailable [15]. To optimize absorption, levothyroxine should be taken on an empty stomach, typically 30–60 min before breakfast. However, this requirement contributes to poor treatment adherence [9]. Food and certain beverages, including soy, coffee, dietary fiber, and papaya, as well as supplements such as iron and calcium, can significantly reduce L–T4 absorption [16]. Additionally, concomitant medications may influence levothyroxine requirements. For example, estrogen therapy increases the need for L–T4 [17]. Due to these factors, approximately 20–50% of patients do not achieve optimal therapeutic response with standard dosing, necessitating ongoing dose adjustments and monitoring [18]. Moreover, this treatment strategy has limitations and may be associated with persistent symptoms, including depression. Therefore, it is crucial to develop novel clinical approaches addressing the underlying autoimmune process of HT [19].

### 3.2. Studies on the Association Between Levothyroxine Treatment and Anti–Thyroid Antibody in Hashimoto’s Thyroiditis

In immunological endocrinology, there is still ongoing debate as to whether levothyroxine can modulate immune mechanisms in the course of Hashimoto’s thyroiditis. This is a controversial topic; it is known that this drug has a favorable effect on women’s hormonal parameters, but there are discrepancies in the results of studies concerning levothyroxine therapy and antibody levels and markers of inflammation in Hashimoto’s thyroiditis. 

#### 3.2.1. Alterations in Antibody Levels During Levothyroxine Treatment

According to the scientific reports, levothyroxine may play a potential role in reducing the levels of anti–thyroid antibodies. Nevertheless, it is crucial to highlight that inconsistencies were observed in the results [14]. Available scientific evidence indicates a reduction in anti–TPO antibody levels by approximately 10–90% following levothyroxine therapy administered for 6–24 months in patients with hypothyroidism [20]. In a retrospective study, Schmidt et al. analyzed anti–TPO concentrations in 36 women and 2 men with Hashimoto’s thyroiditis (initially 111 patients with HT), defined by elevated plasma anti–TPO levels, thyroid hypoechogenicity, and low pertechnetate uptake on thyroid scintigraphy [21]. The study included 17 women and 1 man who did not receive levothyroxine, and 20 patients (19–81 years) who were treated with L–T4. After comparing patients taking levothyroxine at the time of their first visit with those who were not taking it, no significant differences in anti–TPO levels were observed. Each patient underwent up to 8 laboratory tests and neck ultrasound scans (mean = 5.8) over a period of approximately 50 months of L–T4 therapy. The results demonstrated that 35 out of 38 patients (92%) experienced a reduction in anti–TPO levels, with a mean decrease of 8% after 3 months and 45% after one year [21]. Furthermore, five years after the initial measurement, a 70% decrease in anti–TPO levels was observed, and the mean level of these antibodies in the patients studied was 1456 ± 1219 IU/mL. It should be emphasized that, despite the observed decrease in anti–TPO levels in the majority of patients following treatment with L–T4, normalization was achieved in only a small subset of patients after a median follow–up of 50 months. In this study, levothyroxine was found to lower anti–TPO levels, which may have been due to the disease being at a more advanced stage [21]. Significant findings regarding the association between anti-thyroid antibodies and disease duration have also been reported by other authors. Okuroglu et al. investigated the relationship between anti–thyroid antibodies and levothyroxine dose in patients with overt primary hypothyroidism [22]. Importantly, this study found a negative correlation between disease duration and the levels of anti–TPO antibodies (r = −0.159; *p* < 0.05) and anti–TG antibodies (r = −0.021; *p* = 0.765) [22]. The association between L–T4 treatment and levels of anti–thyroid antibodies in hypothyroidism was also demonstrated by Liu et al. in 2019 [23]. They conducted a study involving 29 patients with hypothyroidism (26 women and 3 men) suffering from autoimmune thyroiditis and 18 healthy individuals matched for age and sex. All patients included in the study who were confirmed to have hypothyroidism received levothyroxine. The initial dose was 50 μg/day and was gradually increased until euthyroidism was achieved, with a mean dose of 109.4 μg/day over 12.8 ± 3.2 weeks [23]. Significantly, a decrease in anti–TPO (*p* < 0.01) and anti–TG (*p* < 0.05) antibody levels was observed in patients treated with levothyroxine for hypothyroidism associated with HT. The results obtained by the authors suggest a potential role for L–T4 in ameliorating thyroid destruction in patients with hypothyroidism and HT. Nevertheless, in the final analysis, the study’s limitations—such as the small number of participants, the short follow–up period, and the presentation of only short–term effects of pharmacological treatment—must be taken into account [23]. In 2021, Altun et al. conducted a retrospective study involving 101 women with Hashimoto’s thyroiditis [24]. The data used for the analysis were drawn from hospital databases, and the mean follow–up period was 60.7 ± 32.7 months. Levothyroxine treatment was initiated in patients with TSH ≥ 10 mIU/L or in premenopausal women with TSH between 5–10 mIU/L and elevated thyroid antibodies. At baseline, 34% of patients were euthyroid, 47% had subclinical hypothyroidism, 16% had overt hypothyroidism, and 3% had hashitoxicosis. During follow–up, 30.7% (*n* = 31) of patients remained untreated, while 69.3% received L–T4 (*n* = 70) at a mean dose of 91.5 ± 31.9 µg/day [24]. The average duration of L–T4 therapy in these patients was 60.9 ± 34.3 months. In the group of women taking levothyroxine, the level of anti–TG levels did not alter significantly (*p* = 0.274), whereas anti–TPO levels decreased significantly over time (*p* < 0.001). On the other hand, in the group without levothyroxine, an increase in mean antibody levels was observed, although this was not statistically significant (anti–TPO: *p* = 0.445; anti–TG: *p* = 0.524) [24]. Another study conducted by Syamsundara Kiran et al. involved 106 patients with overt hypothyroidism [25]. Inclusion criteria required levothyroxine therapy for a minimum of 6 months up to 2 years. Patients enrolled in the study continued to take L–T4 for 6 months, after which the therapeutic effects of the treatment on autonomic function, hormonal, inflammatory, and metabolic parameters were assessed [25]. A vital limitation of the study is that only 42 participants completed the 6–month follow–up (32 women and 10 men). A significant reduction in anti–TPO and anti–TG antibody levels was observed in the overt hypothyroid group after 6 months of L–T4 therapy (*p* < 0.001). Despite a substantial drop in anti–TPO and anti–TG titers, they were still significantly high compared to the control group, which consisted of healthy individuals (*p* < 0.001). Consideration should also be given to the lack of data regarding L–T4 dose and its association with anti–thyroid antibody levels [25]. 

In summary, scientific reports have noted a decrease in the level of anti–TPO and anti–TG during treatment with L–T4 in women with hypothyroidism and HT, which may suggest that the drug has an indirect effect on mitigating the humoral immune response and the inflammatory process [21,23,24,25]. Importantly, during levothyroxine therapy, a decrease in anti–TPO antibodies was observed more frequently than in anti–TG antibodies, which showed greater variability [24]. Another aspect requiring attention is the observation of a significant negative correlation between anti–TPO and the duration of HT, which may indicate a decline in antibody levels over time, whether due to the influence of levothyroxine or independently of the drug [22].

#### 3.2.2. Association Between Levothyroxine Dose and Antibody Levels

Analyzing the relationship between levothyroxine treatment and levels of anti–thyroid antibodies in women with hypothyroidism and Hashimoto’s thyroiditis, consideration should also be given to the dose of L–T4. Okuroglu et al. conducted a study involving a population of 273 women and 30 men (19–73 years) [22]. Patients were divided into two groups based on antibody status: antibody–positive (*n* = 210) and antibody–negative (*n* = 93). In the antibody–positive group, the average duration of the disease ranged from 1 year to 34 years, with a mean of 6.2 years. L–T4 pharmacological treatment had been ongoing in patients for at least a year. The mean L–T4 dose was significantly higher in the antibody–positive group (78.83 ± 35.66 μg; *p* = 0.001) compared to the antibody-negative group (64.22 ± 27.013 μg), despite only minor differences in TSH levels between groups [22]. In the antibody–positive group, a weak but statistically significant positive correlation was observed between anti–TPO levels and L–T4 dose (r = 0.217; *p* < 0.01), as well as between anti–TG levels and L–T4 dose (r = 0.158; *p* < 0.05). No significant correlations were found in the antibody–negative group. Taking into account all the results obtained by the authors, higher antibody titers may be associated with increased levothyroxine requirements, but observed correlations were weak and may reflect disease severity rather than a direct causal relationship [22]. On the contrary, a study conducted in 2026 by Szczuko et al. did not confirm the observations described above [26]. 70 Caucasian women with Hashimoto’s thyroiditis diagnosed within the past two years were divided into three groups based on L–T4 dose (≤50 μg, 50–100 μg, >100 μg) [26]. The average dose of levothyroxine taken by patients was 71.16 μg ± 36.15 μg. No significant differences were found in the levels of anti–thyroid antibodies among the diverse groups of patients. The highest mean anti–TPO level was observed in women taking >100 μg/L–T4, and the lowest in patients taking 50–100 μg [26]. On the contrary, the mean anti–TG level was highest in group II (50–100 μg L–T4) and lowest in group III (>100 μg L–T4). It is worth noting that the results revealed no significant association between the dosage of L–T4 and anti–TPO (*p* = 0.0677) and anti–TG (*p* = 0.4258). The study also had limitations that may have affected the obtained results, such as single time point observation, short–term effects, unequal and limited sample size [26]. 

In summary, the effect of levothyroxine dose on antibody reduction remains equivocal, and increasing the dose does not appear to enhance therapeutic outcomes in terms of reducing anti–thyroid antibody levels and inflammation. 

The assessment of risk of bias in the above studies is complex and multifaceted due to the inclusion of various types of studies, including single–arm before–and–after studies, retrospective, and cross–sectional analyses. Most scientific reports included only single–center studies without a control group. Moreover, factors were noted that could potentially bias the conduct and interpretation of the research, such as heterogeneous study groups, short–term observations, and missing information regarding the dose of L–T4 taken by patients, the duration of the disease, and its severity, coexisting autoimmune conditions, medications, and supplements taken. A schematic summary of the studies discussed in this narrative review is presented in Table 1.

### 3.3. Association Between Levothyroxine Treatment and Oxidative Stress

Marchiori et al. evaluated the effects of levothyroxine therapy on biomarkers of oxidative stress and systemic inflammation in patients with hypothyroidism [27]. The study included 17 untreated patients with newly diagnosed primary hypothyroidism due to HT. Following L–T4 treatment at doses of 1.5–1.7 μg/kg/day, significant changes in inflammatory mediators were observed, including an increase in IL–10 (*p* < 0.0001) and decreases in IL–1, IL–6, interferon gamma (IFN–γ), and tumor necrosis factor alpha (TNF–α) (all *p* < 0.0001). No significant change was observed in high–sensitivity C–reactive protein (hs–CRP) levels (*p* < 0.284) [27]. Ates et al. demonstrated that levothyroxine replacement therapy reduces oxidative stress and enhances antioxidant status after 6 months of treatment in patients with hypothyroidism and HT [28]. Free thyroxine (FT4) and anti–TPO levels were identified as independent predictors of oxidative stress parameters [28]. Additionally, a 2025 study confirmed significant reductions in inflammatory markers in patients with overt hypothyroidism following 6 months of levothyroxine therapy [25]. A decrease in malondialdehyde (MDA), which is a marker of lipid peroxidation and oxidative stress (*p* < 0.001), and hs–CRP (*p* < 0.05) was observed, indicating beneficial effects of therapy beyond hormonal normalization [25]. There are also reports in the literature that do not confirm a reduction in oxidative stress among women treated with levothyroxine for hyperthyroidism. In a study conducted in 2025 involving 357 patients with euthyroidism, HT, and treated with levothyroxine, the thiol/disulfide balance, regarded as a marker of oxidative stress, was assessed [29]. Patients treated with L–T4 showed lower concentrations of native and total thiols, as well as higher ratios of disulfide/native thiol and disulfide/total thiol ratios compared to the group not treated with L–T4, which may indicate potential antioxidant depletion and higher levels of oxidative stress [29]. The research findings presented in this narrative review point to the need for further analysis regarding the management of oxidative stress in patients with HT.

### 3.4. Studies on the Combination Therapies and Supplementation in Hashimoto’s Thyroiditis

Studies involving combination therapies were also included in this narrative review. The majority of available studies on supplementation in HT focus on the role of vitamin D. Chahardoli et al. demonstrated a significant reduction in anti–TG antibodies (*p* = 0.009) and TSH levels (*p* = 0.027) in a group of 42 women with HT who received vitamin D supplementation at a dose of 50,000 IU once weekly for 3 months, compared to baseline values [30]. However, no significant reduction in anti–TPO levels was observed compared to the placebo group (*p* = 0.08). Vitamin D supplementation may therefore be beneficial in reducing disease activity in patients with HT, particularly in those with elevated anti–TG antibody levels [30]. Similar conclusions were drawn by other authors, who reported that serum levels of 25–hydroxyvitamin D (25OHD) were positively correlated with thyroid volume (r = 0.145, *p* < 0.001) and negatively correlated with anti–TPO (r = −0.361, *p* < 0.001) and anti–TG (r = −0.335, *p* < 0.001) levels [31]. These findings may suggest a potential role of 25OHD in the development of HT and progression to hypothyroidism [31]. Ostrowska et al. conducted observational and interventional studies in 100 women with HT [32]. All participants received levothyroxine at varying doses, 200 μg/day of selenomethionine, and 30 mg/day of zinc gluconate. Participants were randomized into two groups: group A followed an elimination diet (gluten, eggs, dairy), and group B followed a calorie–matched control diet without elimination. After 6 months, serum TSH levels were significantly lower in group A *(p* < 0.001) [32]. Additionally, significantly greater increases in FT4 and free triiodothyronine (FT3) levels, as well as significant reductions in anti–TPO (*p* = 0.001) and anti–TG (*p* = 0.048), were observed in group A. Women who followed an elimination diet showed significantly better results; however, it is difficult to determine what was due to the change in diet and what was due to the L–T4 treatment and supplementation [32]. Increasing evidence from systematic reviews and meta–analyses suggests a negative correlation between selenium levels and anti–TPO and anti–TG antibody titers in HT [33]. Selenium supplementation may reduce oxidative stress and inhibit inflammatory processes through modulation of the Treg/Th17 balance. Therefore, selenium is increasingly used as an adjunct therapy in chronic lymphocytic thyroiditis [33]. Other studies have shown that combined levothyroxine and selenium therapy provides greater therapeutic benefit compared to levothyroxine monotherapy [34]. In a study of 60 patients, one group received L–T4 alone (*n* = 24), while the other received L–T4 plus selenium (*n* = 36) for 3 months. Significant reductions in anti–TPO (*p* = 0.002) and anti–TG (*p* = 0.015) levels were observed in the combination group [34]. In contrast, in prospective, open–label, quasi–randomized studies, selenomethionine supplementation alone (200 μg/day) reduced only anti–TG levels after both 3 months (*p* = 0.001) and 6 months (*p* = 0.001) [35]. No significant changes were observed in anti–TPO antibody levels, TSH, FT3, FT4, or lymphocyte count. Moreover, the thyroid tissue biopsy revealed no beneficial alterations [35]. Additionally, a study involving 68 patients demonstrated that combined therapy with myo–inositol (600 mg) and selenomethionine (83 μg) resulted in significant reductions in anti–TPO (*p* ≤ 0.001) and anti–TG levels (*p* ≤ 0.001) after six months of treatment [36]. The prevalence of autoimmune thyroiditis appears to decrease with lower iodine intake; however, this finding remains uncertain [37]. Taken all together, combination therapies, including L–T4, vitamin D, selenium, and myo–inositol, may provide additional benefits in treatment and modulation of inflammation and anti–thyroid antibody levels in women with HT.

### 3.5. Studies on the Association Between Levothyroxine and Pregnancy in Women with Hashimoto’s Thyroiditis

The inflammation associated with Hashimoto’s thyroiditis can affect fertility and lead to pregnancy complications. Furthermore, the requirement for levothyroxine in women with Hashimoto’s thyroiditis and hypothyroidism alters during pregnancy and the postpartum period. A meta–analysis by Song et al. (*n* = 28 studies; 8875 participants) demonstrated that women with anti–TPO and/or anti–TG antibodies had a higher risk of miscarriage compared to women without elevated antibody levels [38]. Maraka et al. reported that among 5405 pregnant women with subclinical hypothyroidism, 843 women with a mean TSH concentration of 4.8 mIU/L received levothyroxine [39]. Pregnancy loss was significantly lower among women treated with L–T4 compared to untreated women (*p* < 0.01). Compared with the untreated group, treated women had a lower adjusted risk of pregnancy loss, but a higher risk of preterm birth, gestational diabetes, and preeclampsia. An important conclusion from this study is that treatment of subclinical hypothyroidism may reduce the risk of miscarriage, particularly in women with TSH levels between 4.1 and 10 mIU/L prior to treatment [39]. A meta–analysis conducted by Rao et al. in 2019 demonstrated that levothyroxine therapy was associated with a lower risk of pregnancy loss and preterm birth in pregnant women with subclinical hypothyroidism and autoimmune thyroid disease [40]. In another meta–analysis published in 2025, levothyroxine therapy in women with subclinical hypothyroidism or positive anti–TPO antibodies was associated with a lower risk of pregnancy loss (relative risk (RR) = 0.43; class III evidence), preterm birth (RR = 0.56; class III evidence), and gestational hypertension (RR = 0.63; class IV evidence) [41]. 

During pregnancy, the demand for thyroid hormones increases significantly, necessitating adjustments in levothyroxine dosage. Clinical guidelines generally recommend increasing the L–T4 dose by 20–30% immediately after pregnancy confirmation in women with pre–existing hypothyroidism. However, this approach may not be optimal, and overtreatment remains a concern [42]. In pregnant women with severe subclinical hypothyroidism (TSH > 10 mU/L with normal FT4), initiation of levothyroxine therapy should be considered. In contrast, in euthyroid women with positive anti–TPO antibodies, routine administration of L–T4 is not recommended. During pregnancy, TSH and FT4 levels should be monitored every 4–6 weeks until the 20th week of gestation, and again at approximately 28 weeks [43]. A 2024 analysis involving 1746 pregnant women demonstrated that 37.7% of euthyroid women received levothyroxine, and 22.9% received treatment solely due to autoimmune thyroiditis [42]. Appropriate levothyroxine dosing is essential for preventing adverse maternal and fetal outcomes, including preterm birth and postpartum hemorrhage [42]. In the study by Kiran et al., the median dose of L–T4 in women with hypothyroidism prior to conception was 85.7 mcg/day, and an increase of 14.3 mcg/day (*p* = 0.001) was observed during the first trimester [44]. The increase in levothyroxine dose of 16.7% during the first trimester compared to the preconception period was lower than recommended in international guidelines, which suggest an increase of 30–50%. Furthermore, the study demonstrated that levothyroxine doses in women with autoimmune hypothyroidism were higher both before conception and in the third trimester compared to women without autoimmune disease, which should be considered in future treatment approaches for pregnant women with HT [44].

Postpartum levothyroxine dosing should be individualized. Women with overt hypothyroidism prior to pregnancy should return to pre–pregnancy dosing, whereas discontinuation may be considered in cases of pregnancy–induced subclinical hypothyroidism. TSH should be reassessed 6 weeks postpartum to guide further management [45]. The presented analysis suggests potential benefits of levothyroxine therapy in pregnant women with hypothyroidism and Hashimoto’s thyroiditis; however, additional studies are required to determine the optimal dosing and long–term effects for both mother and child [41]. A summary of studies on the association between L–T4 treatment and pregnancy outcomes is presented in Table 2.

## 4. Factors Influencing Levothyroxine Therapy in Women with Hypothyroidism and Hashimoto’s Thyroiditis

### 4.1. Influence of Physiological Status and Comorbidities on Levothyroxine Dosage

This article also summarizes the key factors influencing the dosing of levothyroxine in women with hypothyroidism and Hashimoto’s thyroiditis, which should be taken into account in future clinical studies and treatment strategies in order to obtain the most accurate information regarding the direct effect of levothyroxine on anti–TPO and anti–TG levels. Levothyroxine requirements depend on body weight, ideal body weight, and lean body mass. As these parameters increase, so does the required dose [17]. Dosing based on lean body mass (LBM) has been shown to be consistent across age groups, BMI categories, and menopausal status. A dose of approximately 2.3 μg/kg LBM may optimize treatment outcomes. However, using actual body weight in obese individuals may lead to overestimation of levothyroxine requirements [46]. A retrospective cross–sectional study involving 586 patients with primary hypothyroidism demonstrated a positive correlation between levothyroxine dose and actual body weight (ABW), but not ideal body weight (IBW) [47]. Additionally, positive antibody status and ABW significantly influenced levothyroxine dosage in multivariate analysis [47]. Additionally, levothyroxine dosage may need to be increased in perimenopausal women due to hormonal changes [17]. Gastrointestinal disorders, food allergies, and intolerances may also necessitate dose adjustments [48]. Another vital consideration is the need to take into account the increased requirement for L–T4 in cases where autoimmune diseases co–occur. In patients with autoimmune polyglandular syndrome type 3 (APS–3), which is the most common type of APS, autoimmune thyroid disease co–occurs with type 1 diabetes mellitus (T1DM) and additional non–glandular autoimmune diseases. It has been shown that these individuals have an increased requirement for levothyroxine compared to cases of HT alone [49]. 

### 4.2. Influence of Gastrointestinal Disorders on Levothyroxine Dosage

Levothyroxine tablets contain a stable sodium salt of L–T4 along with various excipients and require an acidic gastric environment for proper dissolution; disturbances in gastric pH may impair drug absorption [50]. Several gastrointestinal conditions, including gastroesophageal reflux disease, irritable bowel syndrome, food allergies, lactose intolerance, gastritis, inflammatory bowel diseases such as ulcerative colitis and Crohn’s disease, and *Helicobacter pylori* infection, may reduce intestinal absorption of L–T4. In such cases, a levothyroxine absorption test may be useful. Impaired absorption should prompt further gastroenterological evaluation and potential dose adjustment to achieve optimal TSH levels [48]. Autoimmune thyroid disease coexisting with gastric autoimmune disorders is characterized by a distinct cytokine profile. Autoimmune atrophic gastritis is associated with achlorhydria, leading to increased levothyroxine requirements, particularly in patients with gastric atrophy and *Helicobacter pylori* infection [51]. In patients with hypothyroidism and gastritis or *Helicobacter pylori* infection, administration of liquid L–T4 has been shown to improve absorption compared to tablet formulations [52]. Furthermore, a 2024 study demonstrated that lactose intolerance negatively affects levothyroxine absorption, which improves after the implementation of a lactose–free diet. In patients requiring high doses of L–T4 to maintain euthyroidism, evaluation for lactose intolerance, pseudomalabsorption, and other gastrointestinal disorders is recommended [53].

## 5. Areas Requiring Further Research

**Figure 2:**
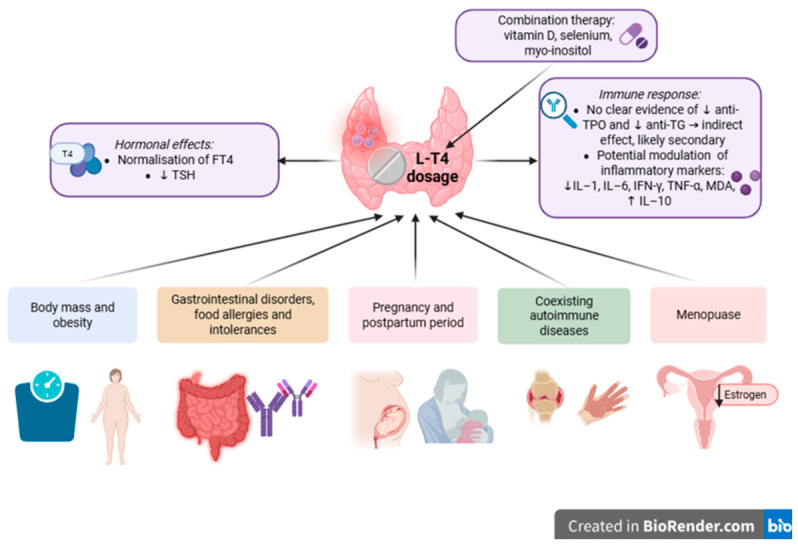
Factors influencing levothyroxine dose in hypothyroidism and Hashimoto’s thyroiditis and the impact of pharmacological treatment on the hormonal–inflammatory axis L–T4—levothyroxine; anti–TPO—anti–thyroid peroxidase antibodies; anti–TG—anti–thyroglobulin antibodies; FT4—free thyroxine; TSH—thyroid–stimulating hormone; IL—interleukin; IFN–γ—interferon gamma; TNF–α—tumor necrosis factor alpha; MDA—malondialdehyde. Created in BioRender. Wrońska K. (2026) https://app.biorender.com/illustrations/673bb726c1abf396d5aa0e79.

All of the above studies emphasize the need for further investigation of the effect of levothyroxine on the levels of anti–thyroid antibodies and fertility in women with HT. The decrease in anti-TPO and anti-TG antibody levels observed in the above studies following levothyroxine treatment may be related not only to the drug-induced suppression of autoimmunity but also to the natural course of the disease; therefore, it is recommended to conduct larger, well-reported, longer-term randomized trials with standardized outcomes and follow-up and repeated measurements. It is also vital to take into account changes in L–T4 dosing depending on the duration and stage of Hashimoto’s thyroiditis, as well as the coexistence of other autoimmune diseases, which may constitute additional factors influencing the therapeutic process. It should also be emphasized that other factors influence the L–T4 dose, such as body weight, body mass index (BMI), pregnancy planning, pregnancy, the postpartum period, gastrointestinal disorders, and concomitant medications or supplements. Further studies should focus on determining the immunological mechanism of action of L–T4, the optimal dose, and the long–term adverse effects of treatment. A summary of the vital factors influencing the levothyroxine dose in hypothyroidism associated with Hashimoto’s thyroiditis and the impact of treatment on the hormonal–inflammatory axis is presented in Figure 2.

## 6. Limitations

It is vital to highlight the limitations of the studies described, such as the varying doses of L–T4 taken by patients, the differing clinical status of the study participants (subclinical hypothyroidism and overt hypothyroidism), variability in the course of the disease, and the presentation of only the short–term effects of L–T4 treatment. Studies examining the relationship between L–T4 treatment and levels of anti–thyroid antibodies in women with Hashimoto’s thyroiditis are very limited and challenging to interpret due to the high variability of factors influencing the therapeutic process in autoimmune diseases. Furthermore, a significant factor influencing the final interpretation of the scientific reports discussed above is the predominance of non-randomized studies. Taken all together, conclusions and clinical recommendations should be formulated with caution. 

## 7. Conclusions

This narrative review summarizes the current knowledge regarding the association between L–T4 treatment and levels of anti–thyroid antibodies in women with Hashimoto’s thyroiditis. In light of the evidence presented in this narrative review, it is impossible to unequivocally confirm that levothyroxine has a direct effect on the decrease in anti–thyroid antibody levels. Based on the available results, increasing the dose of levothyroxine is not associated with improved treatment outcomes in terms of reducing anti–thyroid antibody levels. In addition, the use of combination therapies—including L–T4, vitamin D, selenium, and myo–inositol—in women with hypothyroidism and Hashimoto’s thyroiditis may aid in regulating inflammation and reducing levels of anti–thyroid antibodies. The literature review also confirmed the association between levothyroxine treatment and fertility, as well as improved pregnancy outcomes, in women with hypothyroidism and HT. Future clinical trials should assess whether levothyroxine can also modulate the immune response. Further investigation is warranted to establish therapeutic approaches in women with chronic lymphocytic thyroiditis.

## Figures and Tables

**Table 1:** Summary of studies on the association between levothyroxine and the levels of anti–TPO and anti–TG antibodies in women with hypothyroidism and Hashimoto’s thyroiditis.

Author, Year of Publication	Type of Study	Number of Patients, Gender	Medicines Taken	Results	Comments
Schmidt et al., 2008 [21]	Retrospective study (January 1994–April 2004)	From 111 patients with HT, the analysis was restricted to 36 women and 2 men with a mean age of 51 ± 16 years; 19–81 years	20 patients received L–T4 at a dose that maintained their levels within the normal range	Serum anti–TPO levels decreased in the majority of patients with HT taking L–T4. However, anti–TPO levels turned negative in a small number of patients after a median of 50 months. Only 16% of patients achieved normal anti–TPO levels despite many years of L–T4 treatment.	- No control group - Patients dropout - Varying degrees of disease severity - The selected group of patients - No details regarding the L–T4 doses taken by patients - Assessment of anti–TPO levels only - No detailed information is available regarding comorbidities, diet, other medications, or supplements
Okuroglu et al., 2017 [22]	Retrospective, review of data in medical records (January 2015– December 2015)	303 patients with overt primary hypothyroidism; 273 women and 30 men, aged 19–73	All patients were receiving L–T4 at various doses to maintain appropriate blood TSH levels. L–T4 treatment lasted for at least one year	Hormone replacement therapy to achieve target TSH levels was more common in patients with positive antibodies than in those with negative antibodies. Positive correlation between L–T4 doses and anti–TPO (r = 0.217; *p* < 0.01) and anti–TG doses (r = 0.158; *p* < 0.05) in the antibody positive group.	- The selected group of patients - Varying duration of HT - Higher antibody levels may suggest a more advanced stage of the disease - The correlations found between anti–thyroid antibodies and the L–T4 dose are weak (r < 0.3) - No detailed information is available regarding comorbidities, other medications, or supplements
Liu et al., 2019 [23]	Prospective cohort study	29 hypothyroid drug–naïve autoimmune thyroiditis patients with overt hypothyroidism; mean age: 36.7 ± 9.3; 26 women, 3 men	The initial dose L–T4 was 50 μg/d. The euthyroid state was achieved with a mean dose of 109.4 μg/d.	L–T4 treatment significantly decreased anti–TPO (*p* < 0.01) and anti–TG (*p* < 0.05) in hypothyroid HT patients.	- No comparison with patients with untreated hypothyroidism and HT - The single–center study - The small sample size - Authors presented only short–term effects of L–T4 treatment - Variability in antibody levels
Altun et al., 2021 [24]	Retrospective study (2010–2018)	101 women, mean age at diagnosis was 39.4 ± 12.8 years	31 patients (30.7%) received no treatment and 70 patients (69.3%) received L–T4 treatment; the mean dose was 91.5 ± 31.9 µg/day.	During treatment with levothyroxine, anti–TG levels remained unchanged (*p* = 0.274); however, anti–TPO levels decreased significantly at the time of observation (*p* < 0.001).	- High variability in antibody levels - Varying degrees of disease severity - The lack of information about comorbidities, other medications, or supplements
Syamsundara Kiran et al., 2025 [25]	Follow–up study	106 overt hypothyroid patients–75 women and 31 men. Only 42 participants returned for follow–up after six months–32 women and 10 men.	At baseline, patients were instructed to continue taking L–T4, for six months.	Anti–TPO and anti–TG were significantly decreased after six months of L–T4 therapy in the overt hypothyroid group (*p* < 0.001).	- No comparison with patients with un-treated hypothyroidism and HT - The single–center study - No details were provided regarding the L–T4 doses taken by patients and comorbidities, other medications, or supplements. - The small number of patients completed the study
Szczuko et al., 2026 [26]	Cross–sectional study	70 Caucasian women, 36.68 years ± 8.34 years	Three groups based on their L–T4 dosage: ≤50 μg *n* = 30; 50–100 μg *n* = 30; >100 μg *n* = 10 The average dose of levothyroxine taken by patients was 71.16 μg ± 36.15 μg.	No significant association between the dosage of L–T4 and anti–TPO (*p* = 0.0677) and anti–TG (*p* = 0.4258).	- The unequal and limited sample size - Only Caucasian women - High variability in anti–TG antibody levels - Single–time– point observation

L–T4—levothyroxine; HT—Hashimoto’s thyroiditis; anti–TPO—anti–thyroid peroxidase antibodies; anti–TG—anti–thyroglobulin antibodies; TSH—thyroid–stimulating hormone; *n*—number; r—correlation; *p* value < 0.05—statistically significant.

**Table 2:** L-T4 treatment and pregnancy outcomes in women with hypothyroidism and Hashimoto’s thyroiditis.

Author, Year of Publication	Type of Study	Number of Patients, Gender	Results	Comments
Maraka et al., 2017 [39]	Retrospective cohort study (January 2010–December 2014)	5405 pregnant women with subclinical hypothyroidism; TSH concentration 2.5–10 mIU/L.	A lower risk of miscarriage was observed among women taking L–T4 (*n* = 89; 10.6%) than among untreated women (*n* = 614; 13.5%) (*p* < 0.01). Women receiving pharmacological treatment also had a lower adjusted probability of miscarriage (OR 0.62, 95% confidence interval 0.48–0.82), but a higher probability of preterm birth (1.60; 1.14–2.24), gestational diabetes (1.37; 1.05–1.79) and preeclampsia (1.61; 1.10–2.37).	Incomplete information regarding anti–TPO levels, which made it impossible to carry out a stratification analysis based on the presence of antibodies.
Rao et al., 2019 [40]	Systematic review and meta-analysis (8 studies were randomized controlled trials; 5 studies were retrospective studies)	7970 pregnant women with hypothyroidism and AITD who received L–T4 treatment throughout their pregnancy; in some of these women, levothyroxine treatment was initiated prior to undergoing assisted reproductive technology	L–T4 reduced the rate of pregnancy loss (RR = 0.56, 95% CI: 0.42–0.75, I^2^ = 1%, 12 studies) and the rate of preterm births (RR = 0.68, 95% CI: 0.51–0.91, I^2^ = 21%, eight studies) in women with subclinical hypothyroidism and/or AITD.	The number of studies included in the meta–analysis was limited; larger studies are recommended.
Wang et al., 2025 [41]	Systematic reviews and meta-analyses of randomized controlled trials (11 meta–analyses were included)	11 meta–analyses involving a total of 51,826 pregnant women with subclinical hypothyroidism or anti–TPO positivity; L–T4 treatment before pregnancy and/or started in early pregnancy	L–T4 therapy during pregnancy in women with hypothyroidism was associated with better pregnancy outcomes and a lower risk of miscarriage, preterm birth and gestational hypertension. No significant effect of L–T4 treatment on the rate of live births, placental abruption or gestational diabetes.	Potential biases in selection and reporting.
Kiran et al., 2022 [44]	Retrospective study (2008–2016)	280 pregnant women with hypothyroidism; some of the women had autoimmune hypothyroidism	In women with subclinical hypothyroidism, the dose of L–T4 throughout pregnancy was lower (<100 mcg per day) than in women with overt hypothyroidism. The dose of L–T4 in women with autoimmune hypothyroidism was higher during the preconception period and in the third trimester of pregnancy compared to women without autoimmune disease.	Groups of women that were not of equal size (78 women with autoimmune hypothyroidism and 202 with non–autoimmune hypothyroidism).

L–T4—levothyroxine; AITD—autoimmune thyroid diseases; anti–TPO—anti–thyroid peroxidase antibodies; TSH—thyroid–stimulating hormone; *n*—number; OR—odds ratio; RR—risk ratio; CI—confidence interval; *p* value < 0.05—statistically significant.

## Data Availability

No new data were created or analyzed in this study. Data sharing is not applicable to this article.

## Abbreviations

The following abbreviations are used in this manuscript:

AITD	autoimmune thyroid diseases
anti–TG	thyroglobulin antibodies
anti–TPO	thyroid peroxidase antibodies
CTLA–4	cytotoxic T–lymphocyte–associated protein 4
HT	Hashimoto’s thyroiditis
HLA	human leukocyte antigens
IL1RN	interleukin 1 receptor antagonist
L–T4	levothyroxine
TSH	thyroid–stimulating hormone
